# Case Report: Bilateral optic nerve head masses and retinopathy in a patient with B-cell acute lymphoblastic leukemia receiving CAR T-cell therapy masquerading as fungal endophthalmitis

**DOI:** 10.3389/fimmu.2026.1854882

**Published:** 2026-06-11

**Authors:** Jacob S. Heng, Eugenia Ramos-Davila, Alejandra M. Maiz, Amanda Henderson, Charles G. Eberhart, Paulina Liberman, Jean-Yves Métais, Stephen Gottschalk, Kristen E. Schratz, Heather Symons, Challice L. Bonifant, James T. Handa

**Affiliations:** 1 Retina Division, Wilmer Eye Institute, The Johns Hopkins Hospital, Baltimore, MD, United States; 2 Retina Division, Shiley Eye Institute and Viterbi Family Department of Ophthalmology, La Jolla, CA, United States; 3 Division of Ocular Immunology, Wilmer Eye Institute, The Johns Hopkins Hospital, Baltimore, MD, United States; 4 Neuro-ophthalmology Division, Wilmer Eye Institute, The Johns Hopkins Hospital, Baltimore, MD, United States; 5 Ocular Pathology, Wilmer Eye Institute, The Johns Hopkins Hospital, Baltimore, MD, United States; 6 Department of Bone Marrow Transplantation and Cellular Therapy, St. Jude Children’s Research Hospital, Memphis, TN, United States; 7 Department of Oncology, Sidney Kimmel Comprehensive Cancer Center, Johns Hopkins University School of Medicine, Baltimore, MD, United States

**Keywords:** CAR-T cell therapy, ocular adverse event, optic nerve abnormalities, optic neuropathy, retinopathy, tisagenlecleucel, tumor inflammation, vitreal floaters

## Abstract

Chimeric antigen receptor (CAR) T cells can traffic across tissue planes and thus have considerable potential to clear disease in sanctuary sites inaccessible to alternate systemic treatments. While of great clinical benefit, this unique capability is associated with distinctive potential toxicity. Tumor inflammation-associated neurotoxicity (TIAN) is characterized by local inflammatory toxicity at tumor sites within the CNS, presumably initiated by CAR T cells penetrating the blood–brain barrier (BBB). Specifically, ocular toxicity of CAR T-cell treatment affecting the retina and optic nerve may represent a variant of TIAN and has been infrequently described. We highlight a unique case of bilateral optic nerve head masses and retinopathy masquerading as fungal endophthalmitis in a patient with B-cell ALL treated with CAR T-cell therapy. With early recognition and a multidisciplinary approach, such cases of ocular TIAN can be effectively managed.

## Introduction

Chimeric antigen receptor (CAR) T-cell therapy is effective for extramedullary central nervous system (CNS) disease of B-cell acute lymphoblastic leukemia (B-ALL) and B-cell lymphoma, indicating that CAR T cells can penetrate the blood–brain barrier (BBB). Recently, it has been recognized that such penetration of the BBB by CAR T cells can cause local inflammatory toxicity at tumor sites within the CNS, known as tumor inflammation-associated neurotoxicity (TIAN) (1). TIAN should be distinguished from immune effector cell-associated neurotoxicity syndrome (ICANS), which occurs in 15% to 65% of patients receiving CAR T-cell therapy and is thought to be caused by generalized inflammation-induced CNS dysfunction (2). Ocular toxicities representing a variant of TIAN are an underappreciated and rarely reported adverse event following CAR T-cell therapy (3–11). Here, we describe the case of a pediatric patient undergoing CAR T-cell therapy for refractory B-ALL who developed severe retinopathy with bilateral optic nerve head masses.

## Case description

**Figure 1:**
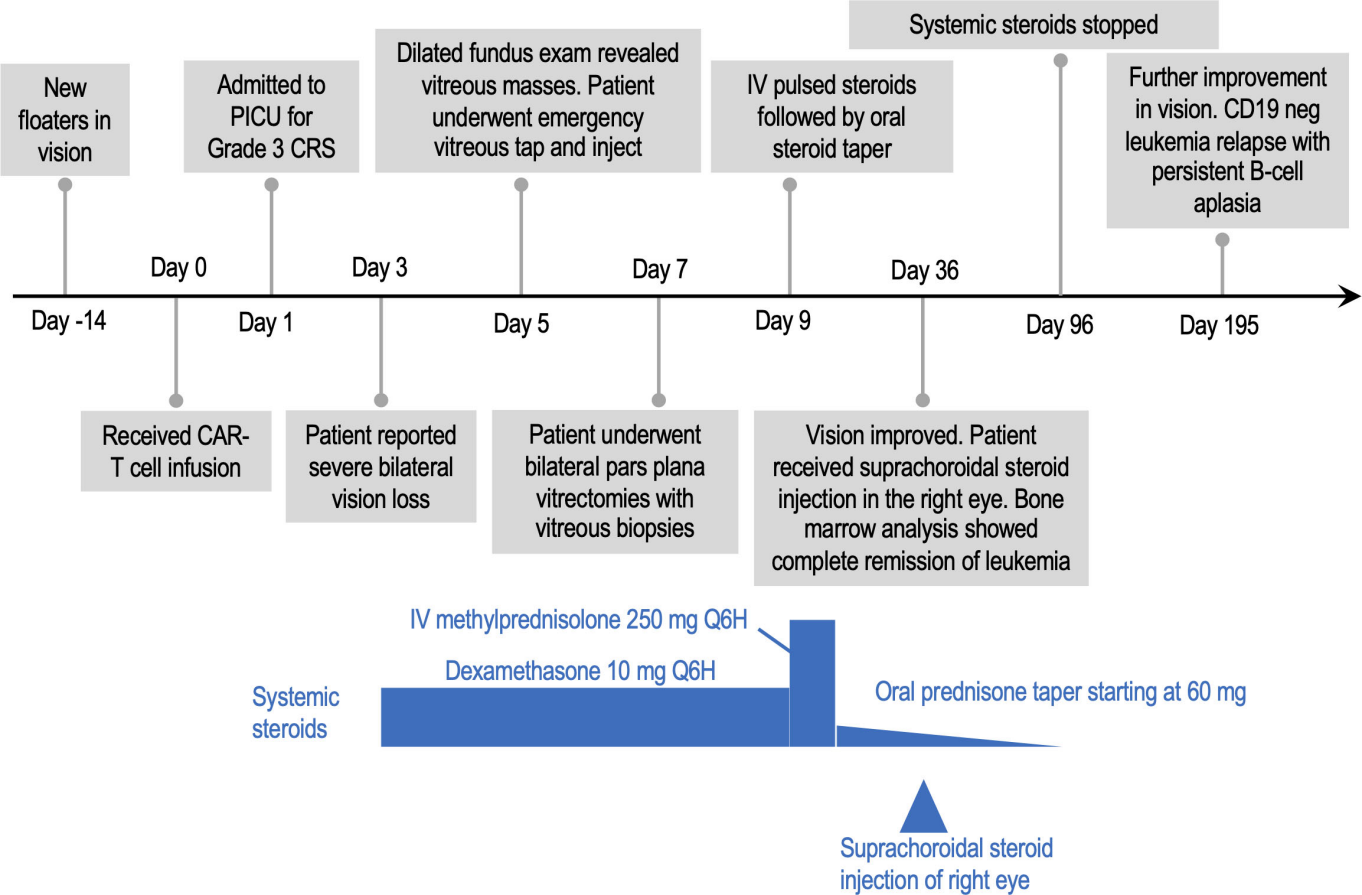
Timeline of the patient’s clinical course. The timeline of the patient’s clinical course, beginning with his initial symptoms of floaters in his vision 14 days prior to CAR T-cell therapy infusion (Day 0) and continuing until relapse of his leukemia despite B-cell aplasia on Day 195, is illustrated.

A 17-year-old male patient with an 8-month history of refractory high-risk CD19+ B-ALL was treated with a standard chemotherapy regimen per the Children’s Oncology Group AALL1732 study protocol, including blinatumomab. He did not have detectable central nervous system or other extramedullary disease and received routine prophylaxis with intrathecal cytarabine and methotrexate. His induction course was complicated by seizures due to an extensive central sinus venous thrombosis, treated with anticoagulation and prompting a postinduction course of blinatumomab, which was well tolerated. Due to persistent low-level marrow disease (0.04%), he received tisagenlecleucel (Kymriah) CAR T-cell therapy following lymphodepletion with fludarabine and cyclophosphamide. His clinical course, beginning around the time that he received CAR T-cell therapy, is illustrated in **Figure 1**. He first reported floaters, but no other visual symptoms, in both eyes 2 weeks prior to receiving CAR T-cell therapy. One day after CAR T-cell infusion, he was admitted to the pediatric intensive care unit (PICU) with grade 3 cytokine release syndrome (CRS), which required vasopressor support and treatment with tocilizumab and IV dexamethasone 10 mg Q6H. Two days into his PICU admission, he developed an altered mental status consistent with grade 1 ICANS. He also reported complete blurring of vision in both eyes, for which the Ophthalmology Service was consulted.

**Figure 2:**
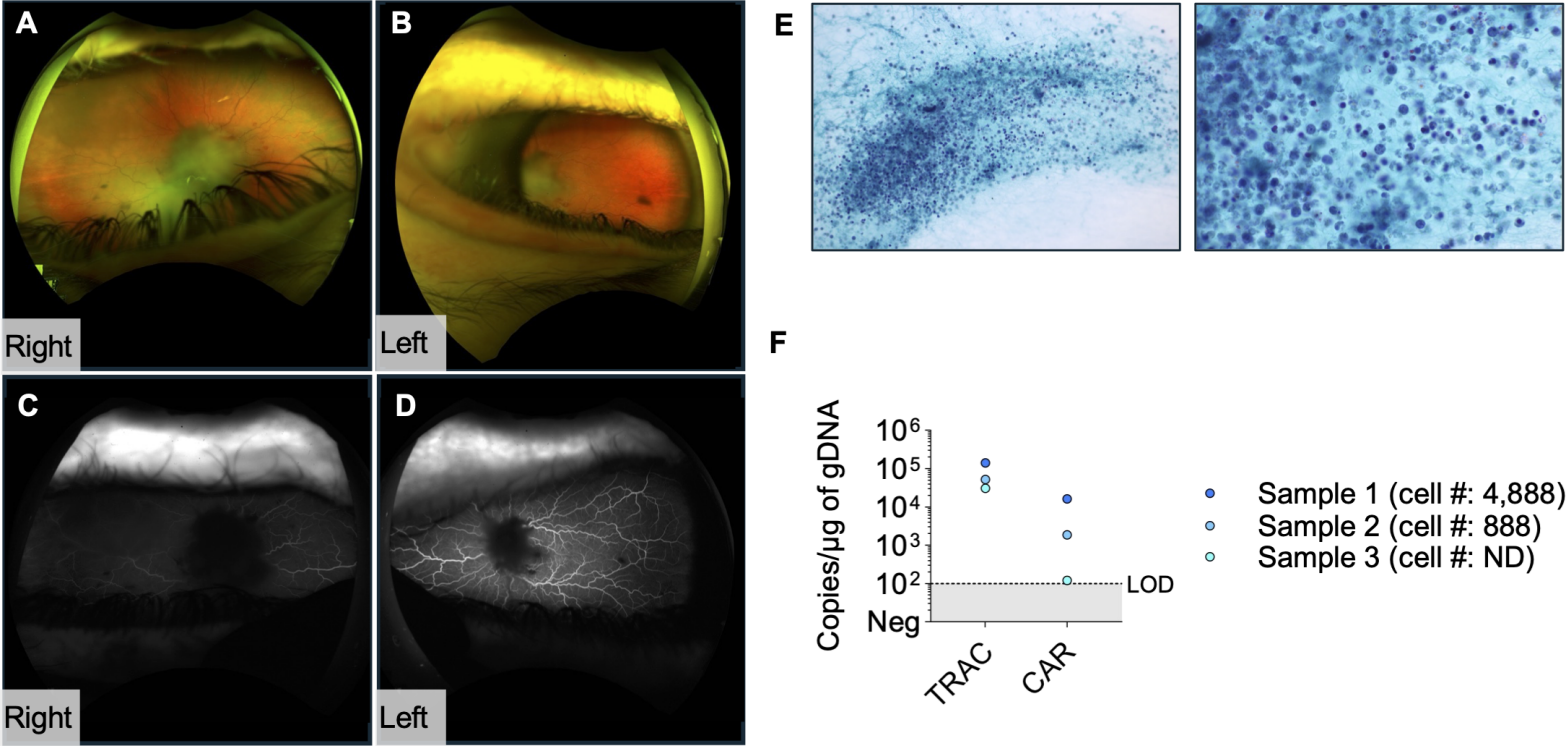
Post-CAR T-cell optic nerve inflammation is associated with CAR T-cell infiltration. **(A)** Widefield color fundus photographs of the right and **(B)** left eye at presentation showing vitreous mass and intraretinal hemorrhages. **(C)** Fluorescein angiogram (FA) of the right and **(D)** left eye showing blocking defect from vitreous masses without apparent vascular leakage. **(E)** Pap-stained smear of a vitreous sample from the right eye at × 200 (right) and × 600 (left) showing scattered lymphocytes and degenerated cells, rare neutrophils, and small histiocytes in a mucoid background. **(F)** Three vitreous biopsy samples from the right eye (sample 1, vitreous mass; sample 2, undiluted vitreous; sample 3, diluted vitreous) were analyzed using a previously published genomic DNA-based qPCR assay to detect the CD19-CAR transgene, with TRAC used as a reference locus for normalization reflecting total cellular DNA input. Limit of detection (LOD) of qPCR assay: 100 copies per μg of genomic DNA (gDNA). ND, not determined; Neg, negative.

On initial examination, visual acuity was counting fingers (CF) at 6 ft in the right eye and 20/200 in the left eye with a relative afferent pupillary defect (RAPD) in the right eye. Dilated fundus examination, which was deferred for 48 h due to the need for frequent neurological status examinations, showed large white fluffy masses in the vitreous cavity occluding the optic disc and macula in both eyes. Due to his history of myelosuppressive chemotherapy and steroid use, a diagnosis of endogenous endophthalmitis was seriously considered. Diagnostic aqueous paracentesis and intravitreal injections of vancomycin, ceftazidime, and amphotericin B were performed in each eye at the bedside. Vitreous taps yielded no vitreous fluid in either eye. The following day, widefield fundus photography showed persistent vitreous masses (**Figures 2A, B**), but fluorescein angiogram (FA) demonstrated no vascular leakage indicative of inflammation (**Figures 2C, D**). Due to the lack of clinical improvement after 36 h, bilateral pars plana vitrectomies with vitreous biopsies were performed. Intraoperatively, the vitreous masses were found to be continuous with the optic nerve head (ONH) and thus carefully excised with the vitrector. All samples of ocular fluids/tissues obtained from his procedures showed no evidence of microorganisms on analysis or culture, including HSV1/2, VZV, CMV, and toxoplasmosis. On cytopathologic evaluation of the vitreous masses, no malignant cells were identified. The sample from the right eye had relatively numerous lymphocytes and degenerated cells, rare neutrophils, and small histiocytes in a mucoid background (**Figure 2E**). The sample from the left eye was virtually acellular. Quantitative polymerase chain reaction (PCR)-based detection of CAR T-cell sequences in vitreous samples (12) was positive (**Figure 2F**). Peripheral blood culture, serum beta-d-glucan, and serum galactomannan were negative, and cerebrospinal fluid examination for blasts was negative.

**Figure 3:**
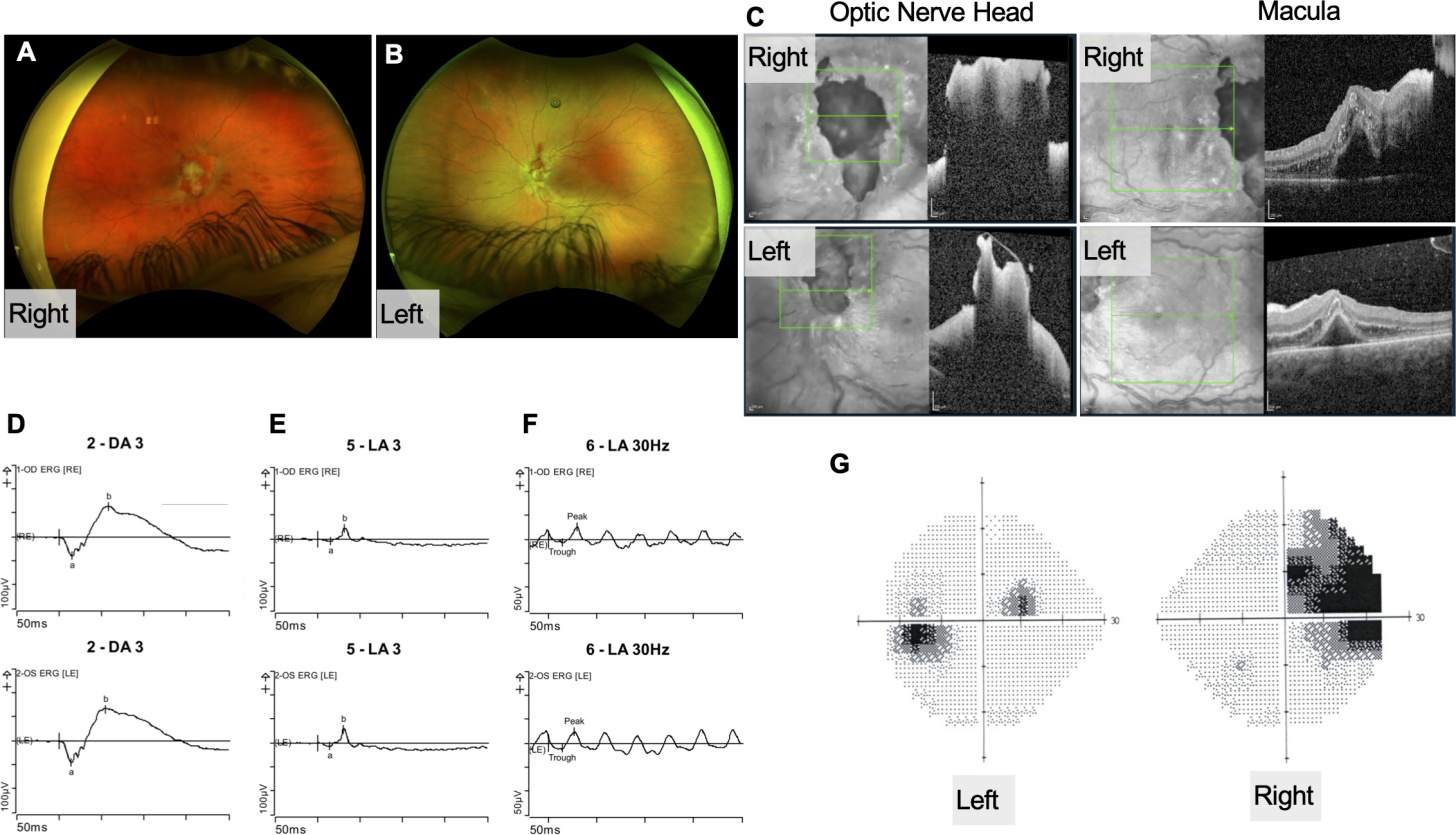
Inflammatory changes and visual deficits persist at 1-month post-CAR T-cell infusion. **(A)** Widefield color fundus photograph of the right and **(B)** left eye at the 2-day post-op follow-up showing attached retina and residual optic nerve head masses with associated optic nerve head swelling and hemorrhages. **(C)** OCTs of the optic nerve head (ONH) at the 2-day post-op follow-up in the right and left eye show mass lesions connected to, but distinct from, the ONH. OCTs of the macula at the 2-day post-op follow-up in the right and left eye show multiple pinpoint hyperreflectivities and intra- and subretinal fluid with concomitant photoreceptor loss. **(D)** Dark-adapted (DA) full-field electroretinogram (ff-ERG) (3.0 cd s/m²) shows slightly reduced a-wave amplitude in both eyes and slightly reduced b-wave amplitude in the right eye. **(E)** Light-adapted (LA) ff-ERG (3.0 cd·s/m²) shows slightly reduced a-wave amplitude in both eyes and slightly reduced b-wave amplitude in the right eye. **(F)** Light-adapted (LA) 30-Hz flicker showing reduced flicker amplitude but normal implicit times. **(G)** Humphrey visual field (HVF) 24–2 showing paracentral scotoma in the left eye and a large nasal visual field defect in the right eye.

Postoperatively and on follow-up evaluation, ONH edema with residual masses at the ONH bilaterally were observed (**Figures 3A, B**). The residual masses appeared to be connected to, but distinct from, the optic nerve heads on ONH optical coherence tomography (OCT, **Figure 3C**). Multiple pinpoint hyperreflective foci possibly representing infiltrating immune cells, macular edema affecting the fovea, and outer nuclear layer (ONL) thinning associated with photoreceptor loss were seen on macular OCT (**Figure 3C**). Leukemic infiltration of the optic nerve was considered in the differential diagnosis, but MRI of the orbits showed no enhancement or abnormality of the retrobulbar optic nerve segments. The overall clinical picture was thus TIAN, which includes massive optic nerve inflammation associated with CAR T-cell infiltration. Therefore, the systemic steroid dosage was increased to IV methylprednisolone 250 mg QID for 3 days, followed by a slow oral prednisolone taper starting at 60 mg daily. The patient also remained on systemic antifungal therapy until fungal cultures were deemed negative after 6 weeks.

At the 1-month postoperative follow-up visit, a full-field electroretinogram (ERG) demonstrated mild generalized dysfunction of the cone photoreceptor system (**Figures 3D–F**) consistent with autoimmune retinopathy (13, 14). A large nasal visual field defect in the right eye and a paracentral scotoma in the left eye were seen on automated visual field testing (**Figure 3G**). Due to the need for continued immunosuppression to treat the residual subretinal fluid, presumed autoimmune retinopathy based on ERG and OCT findings, and optic neuropathy without apparent involvement of the optic nerve segments, the right eye was treated locally with suprachoroidal triamcinolone (Xipere) as oral prednisone was being tapered to avoid long-term systemic steroid exposure. A decision was made to observe the left eye only as systemic steroids were tapered off due to milder disease severity, consistent clinical improvement, and clinical stability. Concurrent bone marrow and CSF analysis revealed a complete remission of leukemia, with no minimal residual disease (MRD) detected either by next-generation sequencing of immunoglobulin rearrangements (sensitivity: 1/1,000,000) or flow cytometry (sensitivity: 1/10,000). Peripheral blood measurement of B-cell aplasia confirmed an absence of B cells and, therefore, functional CAR T-cell persistence.

**Figure 4:**
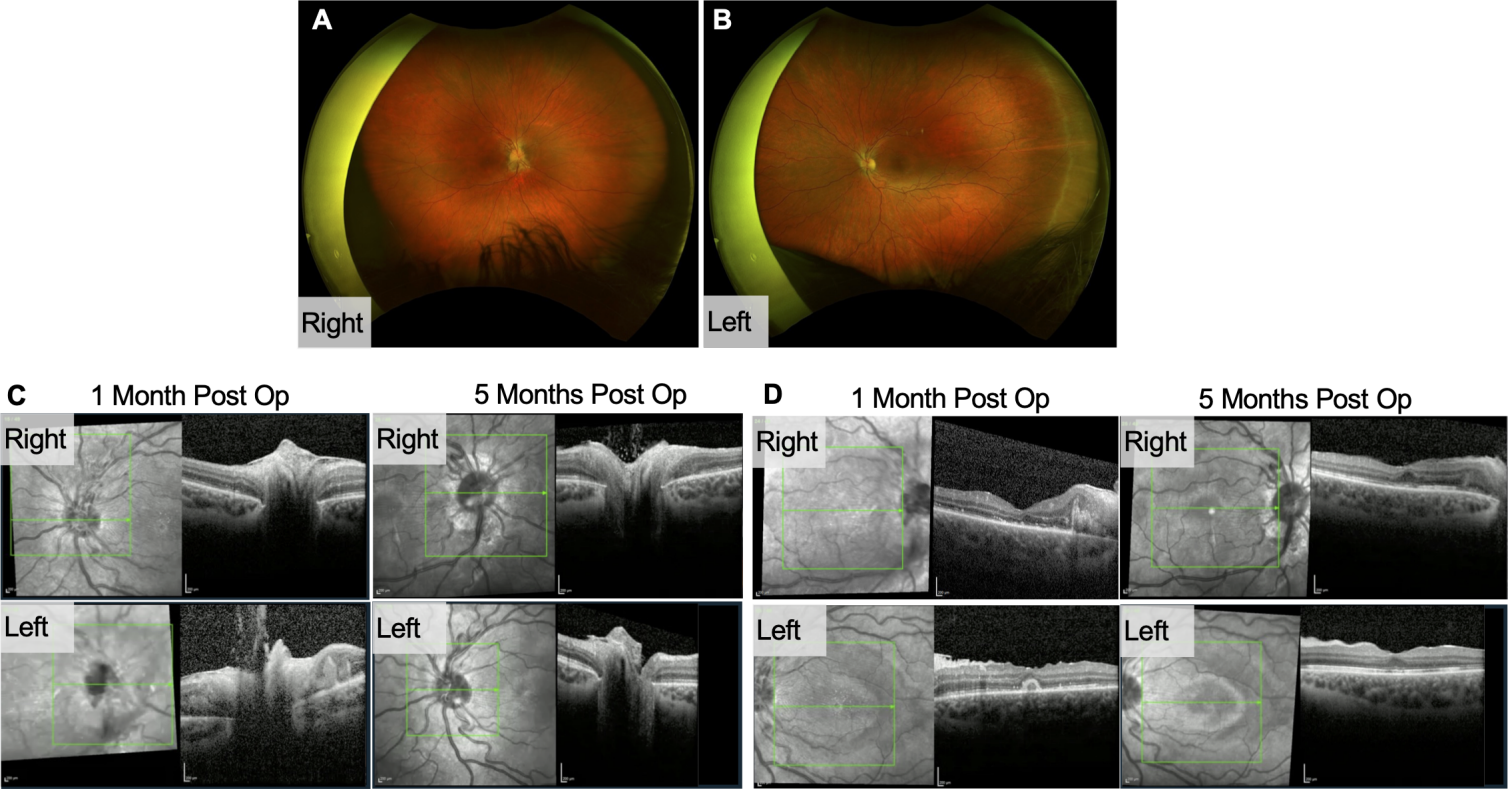
Resolution of optic nerve head masses and macular edema visualized after 5 months of treatment. **(A)** Widefield color fundus photographs of the right and **(B)** left eye at the 5-month post-op follow-up showing an attached retina and no regrowth of vitreous mass. **(C)** OCT of the optic nerve head (ONH) at indicated time points postoperatively shows gradually diminishing residual mass lesions connected to, but distinct from, the ONH bilaterally. **(D)** Serial OCTs of the macula at indicated time points show multiple pinpoint hyperreflectivities associated with intra-and subretinal fluid with concomitant photoreceptor loss bilaterally, with resolution at 5 months.

At 5 months of follow-up, his vision had improved to 20/100 in the right eye and 20/25 in the left eye. B-cell aplasia was persistent. The retina remained attached in both eyes without recurrence of the ONH masses (**Figures 4A, B**) and with resolution of ONH and macular edema in both eyes (**Figures 4C, D**). His B-ALL remained in remission for 218 days when relapse was detected on a surveillance marrow examination at a level of 0.02%. B-cell aplasia was present, and the disease was found to lack CD19 expression.

## Discussion

Ocular adverse events (AEs) of CAR T-cell therapy have been infrequently described and involve the ocular surface, anterior segment, retina, and optic nerve (3–11, 15–17). However, vitreous or optic nerve head masses following CAR T-cell therapy have not been reported. The initial acute presentation of large vitreous masses in the setting of recent chemotherapy in our patient was concerning for fungal endophthalmitis, warranting prompt investigation and treatment. Intraoperative findings and extensive negative microbiological testing indicated that these vitreous masses were, in fact, ONH masses. Our patient also had photoreceptor loss and diminished cone photoreceptor ERG responses consistent with autoimmune retinopathy, previously reported in only one other patient following CAR T-cell therapy (10). Although a dilated fundus examination was not performed prior to our patient’s CAR T-cell therapy to evaluate the pretreatment floaters, it is likely that leukemic cells were already present in the vitreous cavity, which may have infiltrated the optic nerve and retina. These infiltrating cells may have attracted the infused CAR T cells into these structures and recruited reactive inflammatory cells, giving rise to the vitreous/ONH masses and retinopathy. The prominent lymphocytic infiltrate and associated cellular debris seen on cytopathology are morphologically consistent with degenerating CAR T cells, reactive inflammatory cells, and leukemic cells. Although the biopsy material was insufficient to confirm the cells’ identity immunohistochemically, the presence of CAR T cells amongst the cells seen on cytopathology was confirmed by sequencing. Therefore, such focal ocular neurotoxicities are best classified and graded as TIAN, a unique on-target inflammatory toxicity important to distinguish from the generalized neuroinflammation of ICANS (1).

The mainstay of initial treatment for patients with ocular AEs affecting the retina and/or optic nerve from CAR T-cell therapy is systemic corticosteroids. However, the concomitant prolonged use of systemic steroid therapy in patients who have received CAR T cells can be controversial. Short-term (≤ 10 days) steroid use is not associated with any negative prognostic impact (18); however, higher cumulative and prolonged (> 10 days) corticosteroid use is independently associated with early progression and shorter overall survival after CAR T-cell therapy, with median overall survival decreased to 9 months or less (19–21). In our patient, the use of systemic and local steroids did not appear to limit CAR T-cell function, as he maintained B-cell aplasia for > 200 days. Ultimately, his disease relapse at 195 days postinfusion was CD19-negative, reflecting leukemic escape and CD19-CAR T-cell resistance. Although unlikely, it is conceivable that the relatively heavy and prolonged systemic steroid regimen in our patient could have diminished the overall depth of the immune response and facilitated the clonal expansion of CD-19-negative leukemic cells despite the persistence of B-cell aplasia.

## Conclusion

As CAR T-cell therapy is adopted for wider indications, associated ocular AEs are expected to increase. Initial workup and management should include evaluation for infectious etiologies and recurrent malignant disease. Before initiating CAR T-cell therapy, clinicians should conduct a comprehensive review of systems with specific attention to visual complaints and maintain a low threshold for obtaining an ophthalmologic consultation. Although optic nerve infiltration by leukemia may not alter the CAR T-cell treatment plan, it can help guide discussions with families about potential ocular AEs. When ocular AEs, particularly with CRS, affect the retina or optic nerve, systemic corticosteroids remain the mainstay of treatment, although local corticosteroids may also be considered if the retrobulbar optic nerve is not involved.

## Data Availability

The original contributions presented in the study are included in the article/supplementary material. Further inquiries can be directed to the corresponding author.
